# Supplementary material for: Evolutionary and Genetic Recombination Analyses of Coxsackievirus A6 Variants Associated with Hand, Foot, and Mouth Disease Outbreaks in Thailand between 2019 and 2022
Source: Viruses. 2022 Dec 27;15(1):73. doi: 10.3390/v15010073 (PMC9863931; doi:10.3390/v15010073)
Supplement: Supplementary file 1 [file viruses-15-00073-s001.zip › viruses-2093750--supplementary.pdf]

Table S1: Primer set used for VP1 and 3Dpol sequencing

Target region	Primer name	Sequence (5' to 3')	Position*
VP1	CVA6-F2632/VP1	TGT GTG ATG AAT CGA AAC GGG GT	2630-2652
	CVA6-R3288/VP1	TGC AGT GTT AGT TAT TGT TTG GCT	3263-3286
3Dpol	CAV6-F6107-OS	CGC CTC GAG GTR GAT TTY GAR CA	6107-6129
	CAV6-R7428-OAS	CTG GTT ATA ACA AAT TTA CCC CCA CC	7403-7428
	CAV6-F6188/IS	GAR GCA GCH CTV CAY TAT GCA AAY CA	6188-6213
	CAV6-R7388/IAS	CCA GAT TYC TGG TGG GGT TGA G	7367-7388

*Relative to the sequence of the Gdula strain (GenBank accession number AY421764).

<u>Table S2</u>: Primer set used for whole genome amplification by nested RT-PCR

No.	Primer	Sequence (5' to 3')	Position
1	CVA6-F39/OS	ACT GGG CGC YAG CAC ACT GAT TC	39 - 61
	CVA6-R1673/OAS	AGT TAR TGT RAT TGG YAC YTC TGT	1650 - 1673
	CVA6-F55/IS	CTG ATT CTA YGG AAY CTT TGT GCG	55 - 78
	CVA6-R1567/IAS	GGA AGY GCR TTR ACA TAT GGC AT	1545 - 1567
2	CVA6-F1266/OS	TCG GGY TTC TGY ATG CAY GTT CA	1266 - 1288
	CVA6-R2806/OAS	GAY AGT TCT AGY TTG CGC CGC TG	2784 - 2806
	CVA6-F1290/IS	TGY AAY GCR AGC AAR TTC CAT CA	1290 - 1312
	CVA6-R2727/IAS	CCG AGT CCT TYA CCT CCA CAA C	2706 - 2727
3	CVA6-F2458/OS	CRA ATG CDG TGG AAA GYG CTG T	2458 - 2479
	CVA6-R3832/OAS	CCT TTG ATR TAA TCW GAY ACD CC	3810 - 3832
	CVA6-F2485/IS	GCR CTY GCT GAY ACC ACA ATA TC	2485 - 2506
	CVA6-R3816/IAS	GAC ACC CTG YTC CAT RGC TTC	3795 - 3816
4	CVA6-F3498/OS	GCT CAR GGA TGT GAY ACY ATT GC	3498 - 3520
	CVA6-R4564/OAS	CTA GAG TGR TAY TTR TCR GCT AT	4542 - 4564
	CVA6-F3603/IS	GTC TTY GTG GAA GCT AGT GAG TA	3603 - 3625
	CVA6-R4463/IAS	ACG GTG TTT GCT CTT GAA CTG CAT	4440 - 4463
5	CVA6-F4107/OS	AGY GCA TCN TGG CTH AAG AAG TT	4107 - 4129
	CVA6-R5482/OAS	TGA TCY GTY TGV ACY TGC CTR AT	5460 - 5482
	CVA6-F4214/IS	TRT ACC AGC AGC TAA AGA GAA GGT	4214 - 4237
	CVA6-R5330/IAS	GTA GAT RAC ATA CAC CAR TGA RAC	5307 - 5330
6	CVA6-F4994/OS	ATC CAA RGT BAG RTA YAG TGT GGA	4994 - 5017
	CVA6-R6422/OAS	GAG RTC AAR DCC ATA CTT RTC CAT	6399 - 6422
	CVA6-F5061/IS	GCY ATT GGN AAC ACA ATC GAA GC	5061 - 5083
	CVA6-R6364/IAS	GGG TCY AAR ATG TCY CTC TTC TT	6342 - 6364
7	CVA6-F6107-OS	CGC CTC GAG GTR GAT TTY GAR CA	6107-6129
	CVA6-R7428-OAS	CTG GTT ATA ACA AAT TTA CCC CCA CC	7403-7428
	CVA6-F6188/IS	GAR GCA GCH CTV CAY TAT GCA AAY CA	6188-6213
	CVA6-R7388/IAS	CCA GAT TYC TGG TGG GGT TGA G	7367-7388
